# Policy uptake and implementation of the RTS,S/AS01 malaria vaccine in sub-Saharan African countries: status 2 years following the WHO recommendation

**DOI:** 10.1136/bmjgh-2023-014719

**Published:** 2024-04-30

**Authors:** Caroline Bonareri Osoro, Eleanor Ochodo, Titus K Kwambai, Jenifer Akoth Otieno, Lisa Were, Caleb Kimutai Sagam, Eddy Johnson Owino, Simon Kariuki, Feiko O ter Kuile, Jenny Hill

**Affiliations:** 1 Centre for Global Health Research, Kenya Medical Research Institute, Kisumu, Kenya; 2 Department of Global Health, Stellenbosch University, Stellenbosch, South Africa; 3 Centers for Disease Control and Prevention, Kisumu, Kenya; 4 Department of Clinical Sciences, Liverpool School of Tropical Medicine, Liverpool, UK

**Keywords:** Vaccines, Malaria, Child health

## Abstract

In October 2021, the WHO recommended the world’s first malaria vaccine—RTS,S/AS01—to prevent malaria in children living in areas with moderate-to-high transmission in sub-Saharan Africa (SSA). A second malaria vaccine, R21/Matrix-M, was recommended for use in October 2023 and added to the WHO list of prequalified vaccines in December 2023. This study analysis assessed the country status of implementation and delivery strategies for RTS,S/AS01 by searching websites for national malaria policies, guidelines and related documents. Direct contact with individuals working in malaria programmes was made to obtain documents not publicly available. 10 countries had documents with information relating to malaria vaccine implementation, 7 referencing RTS,S/AS01 and 3 (Burkina Faso, Kenya and Nigeria) referencing RTS,S/AS01 and R21/Matrix-M. Five other countries reported plans for malaria vaccine roll-out without specifying which vaccine. Ghana, Kenya and Malawi, which piloted RTS,S/AS01, have now integrated the vaccine into routine immunisation services. Cameroon and Burkina Faso are the first countries outside the pilot countries to incorporate the vaccine into national immunisation services. Uganda plans a phased RTS,S/AS01 introduction, while Guinea plans to first pilot RTS,S/AS01 in five districts. The RTS,S/AS01 schedule varied by country, with the first dose administered at 5 or 6 months in all countries but the fourth dose at either 18, 22 or 24 months. SSA countries have shown widespread interest in rolling out the malaria vaccine, the Global Alliance for Vaccines and Immunization having approved financial support for 20 of 30 countries which applied as of March 2024. Limited availability of RTS,S/AS01 means that some approved countries will not receive the required doses. Vaccine availability and equity must be addressed even as R21/Matrix-M becomes available.

**Summary box**

- The WHO recommended the widespread use of the RTS,S/AS01 malaria vaccine in October 2021 to prevent malaria in children in areas of moderate-to-high malaria transmission.
- The policy has been widely adopted in sub-Saharan Africa with 30 countries expressing interest in rolling out the vaccine; however, only 20 have been approved for support by Global Alliance for Vaccines and Immunization (GAVI).
- Some of the 20 GAVI-approved countries will not receive the required doses due to the limited availability of RTS,S/AS01.
- Two years after the recommendation, the vaccine has been integrated into routine immunisation services in only five countries: Ghana, Kenya and Malawi, where it was piloted, and in two non-pilot countries, Cameroon and Burkina Faso.
- The October 2023 WHO recommendation of the R21/Matrix-M vaccine and its subsequent prequalification in December 2023 will boost vaccine supplies if available as scheduled, by mid 2024.

## WHO recommends RTS,S/AS01 as the world’s first malaria vaccine

Malaria remains a major public health challenge in sub-Saharan Africa (SSA) despite the global gains made in recent years.^1^ In 2022, about 249 million malaria cases were reported, with 94% of cases and 95% of deaths occurring in the WHO African region.^2^ Children are among the most vulnerable populations as they are at higher risk of malaria infection and related complications. While malaria deaths in children aged under 5 years fell from 87% in 2000 to 76% in 2015, progress has stalled, with 78% of all malaria deaths in the WHO African region in 2022 among children under 5 years.^2^

In October 2021, the WHO recommended the RTS,S/AS01 (Mosquirix; GlaxoSmithKline (GSK)) malaria vaccine for the prevention of *Plasmodium falciparum* malaria in children living in regions with moderate-to-high malaria transmission.^3^ This followed more than four decades of basic research and clinical trials.^4^ The RTS,S/AS01 vaccine, extensively tested in different endemic regions in Africa, has moderate efficacy, reducing clinical malaria in children aged 6 weeks to 18 months by 46% (95% CI 41.7% to 49.5%) and severe disease by 36% (95% CI 14.6% to 51.1%) 18 months after the third dose.^5^ Though the effect is short lived and may depend on the transmission intensity, the vaccine also confers protection to HIV-infected children for up to 1 month after the third dose.^6–8^ The WHO recommends a four-dose vaccine schedule with a three-dose primary series given at a minimum interval of 4 weeks between doses in children from 5 months of age, with a fourth dose 12–18 months after the third dose to prolong the duration of protection.^3^ There is a provision for a seasonal five-dose strategy in areas of highly seasonal malaria or with perennial malaria transmission with seasonal peaks, as the HR for the protective efficacy of seasonal vaccination compared with chemoprevention was 0.92 (95% CI 0.84 to 1.01).^3 9^ The vaccine reduced clinical malaria by 46% (95% CI 41.8% to 50.1%) and by 34% (95% CI 28.9% to 38.6%) over a period of 3–4 years when administered with or without the fourth booster dose, respectively.^10^ It has been shown to reduce all-cause mortality (excluding injury) by 13% (0.87, 95% CI 0.78 to 0.98) and hospitalisation due to severe malaria by 22% (0.78, 95% CI 0.64 to 0.96), during the 46 months of its introduction.^11 12^

In 2015, the European Medicines Agency (EMA) gave RTS,S/AS01 a positive recommendation after reviewing the efficacy data from large phase III trials.^10^ Following the EMA recommendation, the WHO recommended its pilot implementation in three countries to determine the impact on the incidence of hospital admission with severe malaria, all-cause mortality and meningitis or cerebral malaria after a possible association was detected in the phase III trials in addition to the feasibility of implementing the recommended four doses in the context of routine health systems.^13 14^ The WHO evaluation was to be conducted over 4 years; however, sufficient data had accrued after 24 months, in October 2021, for WHO to make a recommendation. The evaluation confirmed the vaccine’s safety and demonstrated the feasibility of implementation within the existing Essential Programme on Immunization (EPI) in resource-limited settings.^15^

Despite recent progress with RTS,S/AS01, its efficacy falls well below WHO’s strategic goal set in 2013: ‘By 2030, licensed malaria vaccines should have 75% or higher vaccine efficacy against clinical malaria with duration of protection demonstrated over at least two years’.^16^ A second malaria vaccine, R21/Matrix-M, developed by the University of Oxford, UK and recommended by the WHO for widespread use in October 2023, was the first to achieve the WHO-specified targets with a vaccine efficacy of 78% (95% CI 71% to 83%) in the second year of follow-up following three primary series doses and a booster dose administered seasonally in phase II trials.^17–19^ R21/Matrix-M was added to the WHO prequalified vaccines list in December 2023 and is expected to be available in mid-2024.^12 20^ Results from phase III trials showed R21/Matrix-M reduced clinical malaria cases by 72% (95% CI 69% to 75%) after three primary series doses and a booster dose.^21^ The trial is ongoing, and additional information, such as differences between the standard age-based regimen and seasonal administration after 24 months of follow-up, will be known after quarter 1 of 2024.^22^ The WHO recommends a three-dose primary series of the R21/Matrix-M vaccine given at a minimum interval of 4 weeks between doses in children from 5 months of age, with flexibility on when the fourth dose is delivered, similar to the RTS,S/AS01 regimen.^19^ A fifth dose may be considered in areas of significant risk, and countries may consider providing the vaccine using age-based, seasonal or hybrid delivery strategies in areas with highly seasonal malaria or areas with perennial malaria transmission with seasonal peaks. The WHO recommends that countries prioritise vaccination with RTS,S/AS01 and R21/Matrix-M in moderate and high transmission areas but may also consider providing the vaccine in low transmission settings.^12^ Other vaccine candidates are in development, most in phase I and preclinical stages, with more than 100 ongoing studies as of 2021.^23^ Notably, the *Pf*SPZ vaccine is approaching late-stage clinical evaluation.^3 24 25^

This analysis describes the status of malaria vaccine registration, policy uptake and implementation, two years following the registration of the first vaccine.

## Status of malaria vaccine policy uptake by countries in SSA 2 years on from the WHO recommendation

We conducted an online search for documents reporting on any malaria vaccine (including national malaria guidelines, immunisation schedules, strategic or operational plans for malaria control, government policy papers) from 34 SSA countries with moderate-to-high malaria transmission published between October 2021, the date of the WHO recommendation on RTS,S/AS01, and January 2024. We used the WHO guidance of an annual incidence greater than 250 cases per 1000 population to identify countries with moderate-to-high malaria transmission in 2022.^3 26^ The keywords used in the search included: ‘Malaria’, ‘Guideline’, ‘Immunisation policy’, ‘RTS,S’, ‘R21’ and ‘country’ (where country was the actual name of the SSA country) (table 1). In addition, email contact was made with 24 country representatives working in malaria control programmes to request documents. We relied on members of the Roll Back Malaria partnership on Malaria in Pregnancy and those known to the researchers and were thus unable to contact country representatives from all 34 countries. One subject matter expert, a member of the Independent Review Committee of the Global Alliance for Vaccines and Immunization (GAVI), the organisation responsible for improving access to new and underused vaccines for children, was also consulted for additional information.

**Table 1:** Search approach to identify documents from sub-Saharan countries with moderate-to-high malaria transmission intensity

Search approach	
Databases	Google
Language filter	None
Search terms	‘Guidelines’ OR ‘Immunisation policy’ AND ‘Country’ AND ‘Malaria’ ‘Guidelines’ AND ‘Country’ AND ‘Malaria’ ‘Immunisation policy’ AND ‘Country’ AND ‘Malaria’ ‘R21’ AND ‘Country’ ‘RTS,S/AS01’ AND ‘Country’ ‘R21’ AND ‘Guidelines’ AND ‘Country’ ‘RTS,S/AS01’ AND ‘Guidelines’ AND ‘Country’ ‘RTS,S/AS01’ AND ‘Immunisation policy’ AND ‘Country’ ‘R21’ AND ‘Immunisation policy’ AND ‘Country’

Where ‘country’ is the actual name of the sub-Saharan African country and documents are from October 2021 to January 2024.

Overall, 15 countries had documents or reports concerning the malaria vaccine. The three countries involved in the WHO pilot evaluation (Ghana, Kenya and Malawi) had implemented the vaccine, where expansion of RTS,S/AS01 was ongoing. The pilot countries received financial support from non-profit organisations—GiveWell and Open Philanthropy—through joint fundraising efforts by PATH and WHO—with the required vaccine doses donated by the manufacturer, GSK, and technical and financial support for the pilot evaluations were supported by GAVI, the Vaccine Alliance, The Global Fund and Unitaid. Cameroon and Burkina Faso were the only countries outside of the pilot ones to integrate the malaria vaccine into national immunisation services. Liberia and Benin were yet to implement the vaccine but had received initial RTS,S/AS01 doses. Two other countries (Uganda and Nigeria) had documentation on implementation plans. Through direct contact with individuals, Senegal and Togo were said to be updating their national malaria guidelines to include a malaria vaccine, and Gabon was considering malaria vaccine implementation. A non-governmental organisation (President’s Malaria Initiative) was reportedly providing support for malaria vaccine implementation planning in three countries: Uganda, Burkina Faso and the Democratic Republic of Congo. Togo planned to update their national malaria guidelines in January 2024 following the outcomes of the funding application to GAVI for support for vaccine implementation.

10 countries (Ghana, Malawi, Mali, Uganda, Burkina Faso, Kenya, Nigeria, Cameroon, Benin and Liberia) referred to a specific malaria vaccine, while five countries (Gabon, Guinea, Senegal, the Democratic Republic of the Congo and Togo) did not. Seven countries (Ghana, Malawi, Mali, Uganda, Cameroon, Benin and Liberia) referred to RTS,S/AS01, while three (Burkina Faso, Kenya and Nigeria) referred to both RTS,S/AS01 and R21/Matrix-M. Zambia did not specify a specific malaria vaccine. Burkina Faso, the site of a phase IIb trial of R21/Matrix-M, was among the three countries referring to R21/Matrix-M in addition to Kenya, where the manufacturers of R21/Matrix-M were seeking a licence for deployment in 2023, and Nigeria which had granted R21/Matrix-M conditional licence approval for phase IV trials. Nigeria was reported to have applied for 1 million doses of RTS,S/AS01 to reach 250 000 children, expected to arrive by April 2024. Documents from the remaining 22 countries did not mention either malaria vaccine (online supplemental tables S1 and S2). As of March 2024, Cameroon and Burkina Faso are the first countries outside the pilot countries to introduce the vaccine into national immunisation services, in 42 and 27 districts, respectively.^27–29^ It is noteworthy that the implementation of the malaria vaccine in countries is a rapidly changing situation, and the findings herein offer a snapshot at the time of the study.

10.1136/bmjgh-2023-014719.supp1 Supplementary data

10.1136/bmjgh-2023-014719.supp2 Supplementary data

## Status of RTS,S/AS01 vaccine implementation in each country and strategies that have been deployed

Countries have used varied approaches for introducing the vaccine: (1) pilots with nested evaluations in the three pilot countries—Ghana, Kenya and Malawi (now in the expansion phase, which is restricted to non-vaccinating clusters in the pilot regions due to supply constraints), (2) a planned phased introduction in Uganda with four phases following four subnational categories of districts, based on regions with the highest *P. falciparum* parasite prevalence and high all-cause under-five mortality, scheduled between 2023/2024 and 2026/2027 and (3) a planned pilot programme in five districts in Guinea. The three initial pilot countries and Cameroon use an age-based dosing strategy through routine EPI delivery platforms; however, the RTS,S/AS01 schedule varied across countries. Kenya recommends doses at 6, 7, 9 and 24 months, while Malawi recommends doses at 5, 6, 7 and 22 months. Ghana recommends a schedule of 6, 7 and 9 months for the primary series and the fourth dose at 18 months, changed from 24 months during the vaccine pilot. Cameroon recommends vaccination starts at 6 months, while Benin is to administer it at 6, 7, 9 and 18 months. In Burkina Faso, the vaccine has a four-dose schedule for children starting at 5 months. The variation in scheduling among countries reflects the flexibility the WHO recommendation offers. Other delivery strategies include seasonal vaccination via EPI mass vaccination campaigns (MVCs), a combination of age-based priming vaccination doses delivered via the EPI clinics and seasonal booster doses delivered via MVCs and a combination of age-based priming vaccination doses and seasonal booster doses all delivered via the EPI clinics.^30 31^ However, no country has yet adopted these strategies; only Ghana reported the planned use of mass campaign delivery of RTS,S/AS01 to children aged 6–59 months (online supplemental table S2).

## Limited RTS,S/AS01 availability puts pressure on countries to register non-WHO-prequalified vaccines

30 of 34 SSA countries had expressed interest in implementing the malaria vaccine, as evidenced by their applications to GAVI for financial support. As of March 2024, applications from 20 countries had been approved by GAVI’s Independent Review Committee.^12 32 33^ 18 million doses of RTS,S/AS01 are available for the period between 2023 and 2025.^23^ Of these, 6.9 million doses are needed for the expansion of vaccination in the three pilot countries: Ghana, Kenya and Malawi. The remaining doses will be introduced into routine immunisation in eight countries: Benin, Burkina Faso, Uganda, Cameroon, the Democratic Republic of Congo, Liberia, Sierra Leone and Burundi.^33 34^ Other GAVI-approved countries include the Ivory Coast, Guinea, the Central African Republic, Chad, Nigeria and South Sudan. One country (Niger) will be offered partial supply, while two countries (Mozambique and Sudan) are without immediate allocation due to limited vaccine availability.^33^ The unavailability of vaccines is a major concern, and the problem is expected to get worse as a steady-state demand of approximately 110 million doses annually by 2036 is forecasted.^35^

As witnessed in the recent COVID pandemic, a new vaccine is most likely to be accompanied by a demand that outstrips supply, necessitating protocols that allow its equitable and ethical distribution.^36–38^ To this end, the WHO had rightly developed a framework for vaccine allocation, which considered the principles of transparency, inclusiveness, accountability, greatest need, health impact and equity to prioritise country allocation.^39^ The organisation further advised that the decision on where to introduce the malaria vaccine be made in the context of national planning mixes of malaria interventions and strategies and consideration of subnational tailoring packages of interventions.^26^ Subnational tailoring considers variations in malaria epidemiology, health system structure and function and broader contextual considerations.^26^ Similarly, GAVI proposes the distribution of the available vaccines to countries with areas of greatest need where the proxy measure for need is a composite index of malaria parasite prevalence rates in children and under-five all-cause mortality rates.^34^

However, despite these distribution measures by WHO and GAVI, recommending a life-saving vaccine that was not immediately available for all in need is a concern. Moreover, prioritising the full implementation of available vaccines in the three pilot countries over countries with a greater disease burden is contentious. While it could be argued that the pilot countries have the required vaccine distribution infrastructure and programmes in place to administer limited vaccine supplies, the undeniable burden of disease in other countries is hard to overlook. A trade-off between countries with the greatest need and those that can effectively distribute and administer the scarce vaccine supplies should be considered.^40^

The unavailability of sufficient RTS,S/AS01 vaccine supplies saw countries like Ghana, Nigeria, Burkina Faso and Kenya provide conditional approval of the R21/Matrix-M malaria vaccine at the time when the vaccine was neither WHO recommended nor prequalified.^41^ About 6 million doses of the R21/Matrix-M vaccine are currently available, with the Serum Institute in India projected to produce 100–200 million doses annually.^22^

## Challenges of vaccine introduction

The successful introduction of a new vaccine into national immunisation programmes requires coordinated efforts among all stakeholders. Prior to the WHO recommendation, studies conducted on the anticipated challenges to effective implementation of the vaccine identified inadequate community engagement, fear of vaccine side effects, inefficient delivery of vaccination services to children, cost of vaccine introduction and uptake of the four-dose schedule as possible stumbling blocks.^42–44^ Various approaches have been proposed for the successful implementation of the vaccine, such as using dynamic communication models and trusted sources for delivering vaccine-related health information to the communities, community engagement at both national and district levels and implementation of the new vaccine alongside the existing health services already delivered.^42 43 45–51^

RTS,S/AS01 implementation challenges emerged during the first 2 years in the three pilot countries where the vaccine was incorporated into national EPI programmes. A high dropout rate between the third and fourth doses, 30% in Ghana and Malawi and 40% in Kenya, has been recorded.^52^ While it is recommended that the fourth dose be given 12–18 months after the third dose to prolong the duration of protection, there is flexibility to align the fourth dose with other vaccines given in the second year of life, such as the second measles dose given at 18 months of age. In the three pilot countries, there is flexibility on when the fourth booster dose is administered, ranging from 18 months to 24 months. In Ghana, the provision of the fourth dose in the second year with other vaccines has seen coverage increase to 81%.^53^ Provision of incentives like long-lasting insecticide-treated nets to caregivers and children when they complete the fourth dose is a potential strategy to increase coverage of all four doses.^52 54 55^ Such strategies are needed to integrate RTS,S/AS01 or R21/Matrix-M, another four-dose malaria vaccine, within national EPI schedules.

Vaccine cost is a major consideration for countries implementing the WHO recommendation. The RTS,S/AS01 vaccine is priced at cost with a margin of no more than 5% by the manufacturer, approximately €9.30 per dose, while the R21/Matrix-M vaccine is cheaper at US$4 per dose.^19 22 23^ Funding for the RTS,S/AS01 vaccine for a period of 3 years up to 2025 is provided by GAVI, while UNICEF will secure the supply of the R21/Matrix-M vaccine from 2024 to 2028.^56 57^ A study conducted in the three pilot countries has shown that the cost of introducing the RTS,S/AS01 vaccine is comparable to the cost of introducing other new vaccines, with the country’s baseline immunisation programme capacity a critical factor in the incremental resource needs.^58^ In areas with seasonal malaria transmission, strategies to deliver seasonal doses of malaria vaccines are likely to have a higher cost than age-based EPI delivery.^59^ However, MVCs before the high malaria transmission season, in addition to other intervention measures such as seasonal malaria chemoprevention, are likely to be more effective.^60–62^ It is worth noting that the pilot countries received financial and technical assistance for all components of the pilots (planning, training, communication, vaccines, etc) and for expansion to non-vaccinating clusters. Other SSA countries would benefit from similar support for the implementation of the vaccine through the WHO and GAVI’s Malaria Vaccine Coordination Team, a platform for coordination and information sharing to support the implementation of the vaccine in countries.^12^

## Conclusion

The WHO-recommended RTS,S/AS01 vaccine for children has been well received by countries in SSA, with 30 out of 34 SSA countries approaching GAVI for access to the vaccine and financial support. 20 of the 30 have been approved as of March 2024. Due to supply constraints, implementation of the vaccine within the national immunisation schedules has so far been restricted to the three WHO pilot countries (which also received large grants to scale up) and only two non-pilot countries, Cameroon and Burkina Faso. The limited availability of RTS,S/AS01 when its recommendation for use was made had necessarily prompted the WHO to develop a framework for allocating vaccines, putting some countries on the wait list and thereby putting pressure on countries to register the alternative R21/Matrix-M prior to WHO approval and prequalification. Two years after the much-celebrated WHO recommendation for the RTS,S/AS01 vaccine, there is an urgent need to boost RTS,S/AS01 and R21/Matrix-M vaccine production to meet the demand for these life-saving vaccines in SSA, where the greatest malaria burden lies.
