# Supplementary material for: Policy uptake and implementation of the RTS,S/AS01 malaria vaccine in sub-Saharan African countries: status 2 years following the WHO recommendation
Source: BMJ Glob Health. 2024 Apr 30;9(4):e014719. doi: 10.1136/bmjgh-2023-014719 (PMC11085798; doi:10.1136/bmjgh-2023-014719)
Supplement: Supplementary data [file bmjgh-2023-014719supp001.pdf]

Country of Origin	Type of document								
	National malaria guidelines	National immunization schedule*	National malaria policy	National strategic plan	Newsletter/ News article Press release	Program review	Program operational plan	Therapeutic regimen	**Number of documents (N=80)**
Angola		√					√		2
Benin		√		√	√				3
Burkina Faso	√	√					√		3
Burundi		√		√					2
Cameroon		√		√	√√√				5
Central African Republic		√							1
Chad		√							1
Congo		√							1
Democratic Republic of Congo		√					√		2
Equatorial Guinea		√							1
Ethiopia		√		√					2
Gabon		√							1
Gambia		√							1
Ghana		√	√	√ √	√				5
Guinea		√		√					2
Guinea Bissau		√							1
Ivory Coast	√	√		√ √				√	5
Kenya		√ √			√				3
Liberia		√			√				2
Madagascar		√		√					2
Malawi		√		√	√ √ √				5
Mali		√		√			√		3
Mozambique		√					√		2
Niger		√					√		2
Nigeria		√		√	√ √		√		5
Rwanda		√				√			2
Senegal	√ √	√							3
Sierra Leone		√							1
South Sudan		√			√				2
Tanzania		√					√		2

Togo		√							1
Uganda		√			√		√		3
Zambia		√		√		√			3
Zimbabwe		√							1
**Total**	4	35	1	14	9	2	9	1	80

***34 of the immunisation schedules obtained from the WHO immunization dashboard; one (Kenya) obtained from the Ministry of Health website. Empty cells in the table document not obtained from search or direct contacts.*

Table S1: Characteristics of documents
