# Supplementary material for: Policy uptake and implementation of the RTS,S/AS01 malaria vaccine in sub-Saharan African countries: status 2 years following the WHO recommendation
Source: BMJ Glob Health. 2024 Apr 30;9(4):e014719. doi: 10.1136/bmjgh-2023-014719 (PMC11085798; doi:10.1136/bmjgh-2023-014719)
Supplement: Supplementary data [file bmjgh-2023-014719supp002.pdf]

Table S2: Status and plans of malaria vaccine roll-out by country

Country	Type of vaccine			Approach for vaccine introduction	Vaccine schedule	Mass campaign	Approved by GAVI for support**	Roll-out plans	Additional information (Source)*
	RTS,S/ AS01	R21	Both						
Benin	√				6, 7, 9 and 18 months		Yes	Yes	First vaccinations expected to take place within afew months following the January 2024 delivery of 215, 900 RTS,S/AS01 doses. https://www.voaafrica.com/a/benin-receives-first-doses-of-malaria-vaccine/7441896.html
Burkina Faso			√	Not mentioned	4 doses starting at 5 months		Yes	Yes	Following the promising results from a Phase 2b trial o f the R21 malaria vaccine in Nanoro, Burkina Faso, PMI will support the MoH to develop a vaccine roadmap a nd timeline. (U.S. President's Malaria Initiative: Burkina Faso Malaria Operational Plan FY 2023)
Cameroon	√			Integration into immunisatio n program	Starts at 6 months		Yes	Yes	The vaccine is being rolled out across 42 health districts in the country's 10 regions in public and private health facilities. The launch comes after Cameroon received 331 200 doses of the vaccine in November 2023. 1. https://www.who.int/news/item/22-11-2023-shipments-to-african-countries-herald-final-steps-toward-broader-vaccination-against-malaria--gavi--who-and-unicef

									2. https://www.afro.who.int/countries/cameroon/news/cameroon-kicks-malaria-vaccine-rollout<br>3. https://www.statnews.com/2024/01/22/gsk-malaria-vaccine-mosquirix-rollout-africa/<br>4. https://www.statnews.com/2024/01/22/gsk-malaria-vaccine-mosquirix-rollout-africa/
Democratic Republic of Congo							Yes	Yes	PMI plans to support the introduction of the malaria vaccine into health facilities through the reinforcement of healthcare workers' capacity, supportive supervision, malaria data (including vaccine administration) monitoring (U.S. President's Malaria Initiative, Democratic Republic of the Congo Malaria Operational Plan FY 2023)
Gabon							No		Do not have a plan yet but are considering the introduction of the malaria vaccine in the 2023-2026 Malaria strategic plan (Direct contact with a country representative)
Ghana	√			Pilot and full integration into immunisation program	6, 7, 9 and 18 months	Yes, for children 6-59 months	Yes		1. https://immunizationdata.who.int/pages/schedule-by-country/gha.html?DISEASECODE=&TARGETPOP_GENERAL=<br>2. https://d1u4sg1s9ptc4z.cloudfront.net/uploads/2023/01/FY-2023-Ghana-MOP.pdf<br>3. https://www.malariavaccine.org/news-events/news/phased-expansion-malaria-vaccine-use-begins-more-areas-africa<br>4. https://www.who.int/docs/default-source/immunization/mvip/framework_for_policy_decision_on_rtss-as01_malaria_vaccine.pdf?sfvrsn=ce4d2025_8)

									5. National guideline from direct contact with a country representative)
Guinea							Yes		Are planning a pilot in 5 districts – Kankan, Yomou, Mamou, Gaoual and Siguiri. Full integration into immunisation program planned after pilot. The vaccine schedule planned is 6,7,9 and 18 or 22 or 24 months.<br>(Direct contact with a country representative)
Kenya			√	Pilot and full integration into immunisation program	6, 7, 9 and 24 months		Yes		1. https://www.health.go.ke/)<br>2. https://www.the-star.co.ke/news/2022-04-25-malaria-vaccine-being-tested-in-kenya-enters-homestretch/<br>3. https://immunizationdata.who.int/pages/schedule-by-country/ken.html?DISEASECODE=&TARGETPOP_GENERAL=)
Liberia	√			Not mentioned			Yes		112,000 RTS,S/AS01 doses delivered in January 2024. Ministry of health of Liberia plans to start vaccination in April 2024.<br>https://www.afro.who.int/countries/liberia/news/ministry-health-gavi-who-and-unicef-announce-arrival-112000-doses-malaria-vaccine-liberia
Malawi	√			Pilot and full integration into immunisation program	5, 6, 7 and 22 months		Yes		1. https://immunizationdata.who.int/pages/schedule-by-country/mwi.html?DISEASECODE=MALARIA&TARGETPOP_GENERAL=<br>2. https://www.who.int/docs/default-source/immunization/mvip/framework_for_policy_decision_on_rtss-as01_malaria_vaccine.pdf?sfvrsn=ce4d2025_8

									3. https://www.who.int/news-room/feature-stories/detail/mothers-in-malawi-value-the-first-malaria-vaccine<br>4. https://www.who.int/news/item/20-04-2021-rts-s-malaria-vaccine-reaches-more-than-650-000-children-in-ghana-kenya-and-malawi-through-groundbreaking-pilot-programme<br>5. https://www.malariavaccine.org/news-events/news/phased-expansion-malaria-vaccine-use-begins-more-areas-africa)
Mali	√			Not mentioned			No	Yes	National malaria strategic plan 2023-2027 -yet to be published (Direct contact with a country representative)
Nigeria			√	Not mentioned			No	Yes	Applied for 1 million doses of the RTS,S/AS01 vaccine to reach 250,000 children. Expected in the country by 2024. Conditional approval of the R21 vaccine to be used in phase 4 trials in the country.<br>(Statement on malaria vaccine in Nigeria shared by Direct contact with a country representative)
Senegal							No		Currently updating guidelines to include the vaccine (Direct contact with a country representative)
Togo							**No**		Hope to update the national guideline in January 2024 following a funding application. (Direct contact with a country representative)
Uganda	√			Phased implementation			Yes	Yes	Phased introduction due to limited vaccine supply. Phase 1 in 2023/24. Four phases were scheduled between 2023/24 and 2026/27 according to four sub-national categorisation of the districts. (Http://library.health.go.ug/communicable-disease/malaria/national-malaria-control-division-news-letter)
Zambia				Not mentioned			No	Yes	(https://static1.squarespace.com/static/58d002f017bffcf99fe21889/t/63bd160c30c0dc5ead80cfdb/1673336

									347465/Zambia+Malaria+Programme+Review+2021_SIGNED+120722.pdf)

* Data obtained from websites and direct contact with country representatives; Websites last accessed January 30, 2024. Empty cells denote information not obtained from search and direct contacts.

Documents obtained for the following countries made no mention of the vaccine: Angola, Benin, Burundi, Cameroon, Central African Republic, Chad, Congo, Equatorial Guinea, Ethiopia, Gambia, Guinea Bissau, Ivory Coast, Liberia, Madagascar, Mozambique, Niger, Rwanda, Sierra Leone, South Sudan, Zimbabwe. **Other countries not on the table but approved by GAVI for support in the roll-out of the vaccine as of March 2024: Chad, Central African Republic, South Sudan, Sierra Leone, Burundi, Niger (partial supply), Mozambique and Sudan (both countries have no immediate allocation due to limited vaccine availability), and Ivory Coast.
